# Higher Lipopolysaccharide Binding Protein and Chemerin Concentrations Were Associated with Metabolic Syndrome Features in Pediatric Subjects with Abdominal Obesity during a Lifestyle Intervention

**DOI:** 10.3390/nu13020289

**Published:** 2021-01-20

**Authors:** Amelia Marti, Isabel Martínez, Ana Ojeda-Rodríguez, María Cristina Azcona-Sanjulian

**Affiliations:** 1 Department of Nutrition, Food Sciences and Physiology, University of Navarra, C/Irunlarrea, 31008 Pamplona, Spain; isamartinezdominguez@gmail.com (I.M.); aojeda.5@alumni.unav.es (A.O.-R.); 2 IdiSNA, Instituto de Investigación Sanitaria de Navarra, C/Irunlarrea, 3, 31008 Pamplona, Spain; cazcona@unav.es; 3 Biomedical Research Centre Network on Obesity and Nutrition (CIBERobn), Physiopathology of Obesity and Nutrition, Institute of Health Carlos III, Av. Monforte de Lemos, 3–5, 28029 Madrid, Spain; 4 Paediatric Endocrinology Unit, Department of Paediatrics, Clínica Universidad de Navarra, Av. Pío XII, 36, 31008 Pamplona, Spain

**Keywords:** lifestyle intervention, obese children, chemerin, metabolic syndrome, lipopolysaccharide binding protein

## Abstract

**Background:** Elevated circulating plasma levels of both lipopolysaccharide-binding protein (LBP) and chemerin are reported in patients with obesity, but few studies are available on lifestyle intervention programs. We investigated the association of both LBP and chemerin plasma levels with metabolic syndrome (MetS) outcomes in a lifestyle intervention in children and adolescents with abdominal obesity **Methods**: Twenty-nine patients enrolled in a randomized controlled trial were selected. The lifestyle intervention with a 2-month intensive phase and a subsequent 10-month follow-up consisted of a moderate calorie-restricted diet, recommendations to increase physical activity levels, and nutritional education. **Results:** Weight loss was accompanied by a significant reduction in MetS prevalence (−43%; *p* = 0.009). Chemerin (*p* = 0.029) and LBP (*p* = 0.033) plasma levels were significantly reduced at 2 months and 12 months, respectively. At the end of intervention, MetS components were associated with both LBP (*p* = 0.017) and chemerin (*p* < 0.001) plasma levels. **Conclusions**: We describe for the first time a reduction in both LBP and chemerin plasma levels and its association with MetS risk factors after a lifestyle intervention program in children and adolescents with abdominal obesity. Therefore, LBP and chemerin plasma levels could be used as biomarkers for the progression of cardiovascular risk in pediatric populations.

## 1. Introduction

The worldwide prevalence of overweight and obesity in children and adolescents aged between 5 to 19 years has increased from 4% to 18% over the past four decades [1]. Current estimates suggest that the prevalence of obesity will increase to 25% in children by 2050 [2]. Furthermore, one of the main problems of childhood obesity is that it usually persists into adulthood [2,3].

Furthermore, the chronic and low-grade inflammation linked to obesity is promoted by the abnormal secretion of adipokines and acute phase proteins that exert proinflammatory actions. Circulating inflammatory molecules are considered potential inducers of susceptibility to obesity-related disorders and metabolic syndrome (MetS) features. Thus, some adipokines and acute phase proteins such as CRP, IL-6, TNF-α, and leptin [4], have been widely studied and are considered to be proinflammatory biomarkers of obesity and MetS progression in children and adolescents.

The discovery of biomarkers is useful in the early detection of MetS as well as complications of obesity [5]. Obesity induces a systemic inflammatory state that determines dysfunction of macrophages and adipocytes and, therefore, inappropriate cytokine production. As of yet, no biomarker profile has been found to distinguish patients at greater risk for obesity-related diseases. The usefulness of a biomarker could be extended to guide pharmacological and non-pharmacological therapeutic interventions. It is important to search for a marker with predictive relevance, which could detect cardiovascular risk in MetS early to safeguard the population from the adverse effects of these diseases in early life [6].

Notably, circulating inflammatory biomarkers including plasma levels of both lipopolysaccharide-binding protein (LBP) and chemerin have been reported in patients with obesity. Chemerin is an adipokine mainly produced in adipose tissue involved in the differentiation of adipocytes [7]. It functions as a chemoattractant for immune cells and also affects the metabolism of carbohydrates and lipids. Whether chemerin is a pro- or anti-inflammatory protein is still a matter of debate. In cross-sectional studies, a relationship between chemerin and anthropometric measures or features of MetS was reported in both adult [7,8] and children [9,10,11,12,13].

LBP is a 65-kDa soluble acute phase protein, mainly produced by hepatocytes, and also expressed and released by both intestinal epithelial cells and visceral adipocytes. High LBP levels in white adipocytes are associated with white adipose tissue dysfunction in obesity. Plasma LBP levels increase greatly in response to proinflammatory challenges, such as energy-rich foods, a high-fat diet, sedentary lifestyle and stress [14,15,16]. In cross-sectional studies with adult and young obese subjects, increased LBP plasma concentrations are related to components of MetS [17].

Nevertheless, to our knowledge, there are few studies evaluating the role of these cytokines in lifestyle inventions aimed at weight loss [18]. Thus, the aim of this study was to investigate the association of both LBP and chemerin levels with MetS outcomes after a lifestyle intervention in children and adolescents with abdominal obesity.

## 2. Subjects and Methods

The IGENOI study (Intervention of Grupo Estudio Navarro de Obesidad Infantil) is a randomized controlled trial (ClinicalTrials.gov, Identifier: NCT03147261) consisting of a 2-year outpatient program based on the Mediterranean diet, physical activity, and nutritional education [19,20,21]. Participants (aged 7 to 16 years) were recruited from the Paediatric Endocrinology Units at both *Clínica Universidad de Navarra* and *Complejo Hospitalario de Navarra*, in Pamplona, Navarra, Spain.

Subjects with a history of diabetes, with other diseases apart from obesity, or major psychiatric illness including bulimia nervosa, and those receiving pharmacological treatment, or with food intolerances, or those under treatment with special diets, or frequently consuming alcohol or drugs were excluded. The study protocol was performed in accordance with the ethical standards of the Declaration of Helsinki, and was approved by the Ethics Committee of the University of Navarra (Reference Number 143/2014). Written informed consent was obtained from all children and their parents.

### 2.1. Lifestyle Intervention

The multidisciplinary intervention consisted of a two-year program that comprising a 2-month intensive phase with individual and group sessions and a follow-up period at 10 and 22 months. A multidisciplinary team, including registered dieticians, pediatricians, physical activity experts, and nurses, carried out the intervention in a clinical setting. Parents or legal guardians accompanied the participants in the visits.

Subjects were randomly assigned to the usual or intervention group. The randomization was performed using computer-generated randomization. The usual care group received one 30-min individual session with the dietitian and received standard paediatric recommendations for a healthy diet, and a total of 10 monitoring visits to assess anthropometric measurements during the first year of intervention. During the 2-month period, usual care subjects and their parents received a 30 min individual session with the dietician and five monitoring visits to assess anthropometric parameters.

The intervention group was advised to follow a full-day meal plan during the intensive phase, consisting of a moderately hypocaloric Mediterranean diet. The dietary pattern was based on a high consumption of fruits (3 portions per day) and vegetables (2 portions per day), legumes, whole grains, and olive oil; moderate consumption of dairy products, poultry, and fish, and the reduction of processed and red meats, limiting these to 1 portion per week. The calorie restriction applied ranged from 10–40% depending on the standard deviation score of the Body Mass Index (SDS-BMI) while at the same time attempting to not interfere with the participants’ body growth. During the 2-month intensive phase, patients were prescribed to follow-up the diet and they received six 30-min sessions, every two weeks conducted by the dietitian in order to evaluate the compliance with the diet and to take anthropometric measurements. In addition, they received one parallel group session where the children and adolescents learnt about healthy lifestyles including eating behavior (portion size) and the importance of being physically active (sedentary activities and physical activity). During the follow-up period, the intervention participants received monitoring visits at 3, 4, 5, 6, 9, and 12 months, where nutrition educational topics about healthy breakfast and food choices were given at months 3 and 5 from baseline. Also, at month 4, a group session about groups of foods and frequency were taught to the children and parents or legal tutors.

Regarding physical activity, participants were advised to accumulate 200 min of physical activity per week at 60–75% of their maximum heart rate as recommended by The American College of Sports Medicine. Detailed information about the lifestyle intervention has been published elsewhere [19,20,21].

In total, twenty-nine children (14 boys, 15 girls) who participated in the IGENOI trial and had plasma samples available at the three time points were included in the present study. No statistically significant differences in the anthropometric or biochemical variables of interest were found in participants from the usual care and the intervention group in this subsample.

### 2.2. Anthropometric, Clinical and Biochemical Measurements

All anthropometric and biochemical measurements were carried out at baseline, after the 2- and 12-month interventions using validated procedures. Measurements were performed by trained personnel; all participants were barefoot and wore light clothing. All measurements were assessed three times using the mean as the final data value. Height was measured to the nearest centimeter using a rigid stadiometer (Seca^®^ 220, Vogel & Halke, Hamburg, Germany). Weight and body composition were estimated by bioelectrical impedance analysis (BC-418 TANITA Uxbridge, UK). BMI was calculated as weight in kilograms (kg) divided by the square of height in meters (m^2^), BMI-SDS was calculated using local reference BMI standards, which is adjusted for sex and age [22,23]. Waist circumference (WC) was measured half-way between the lowest rib and the iliac crest and hip circumference (HC) was measured at the widest part of the hips. The WC (cm) to height (H, cm) ratio (WHR) was calculated. Pubertal development was assessed according to Tanner stage [24]. Blood pressure was measured using a validated protocol. Systolic and diastolic blood pressure (SBP and DBP) were measured twice, in the non-dominant arm, after a 15-min rest by using a calibrated sphygmomanometer. Venous blood samples were obtained by specialized trained nurses, at the medical centers after overnight fasting. Glucose, insulin, lipid profile, and leptin were determined in plasma samples by standard autoanalyzer techniques. Plasma LBP levels were measured by a human LBP enzyme-linked assay (ELISA) kit (RayBiotech, Norcross, Georgia) according to the kit instructions. The samples were diluted 1/1000. Plasma chemerin levels were measured with the “human Chemerin sandwich-type ELISA” (Human chemerin ELISA, BioVendor, Brno, Czech Republic) according to the kit instructions. The samples were diluted 1/100. The sensitivity assay yields 4.67 pg/mL.

### 2.3. Statistical Analyses

STATA for Windows 12.0 software (StataCorp, CollegeStatio, Texas, EEUU) was used for statistical analyses. The level of statistical significance was *p* < 0.05. The results are expressed as mean ± standard deviation or median and interquartile range, if not normally distributed. The Shapiro-Wilk test was used to determine the distribution of the variables. Unpaired *t*-test was performed to assess parametric data differences between groups (usual care and intervention) at baseline. Since there were no statistically significant differences in the anthropometric and biochemical variables of interest between groups (usual care and intervention group), the analysis was performed on the whole sample of subjects. The outliers of LBP and chemerin were removed. Repeated measures ANOVA and Tukey Post-Hoc Test, two-sided Student’s *t*-test for unpaired and paired observations, Duncan test, Cochran test, and p-trend test were also used as appropriate. Pearson’s correlation coefficient was used to assess the statistical relationship between continuous variables. We also fitted simple linear regression analyses to evaluate associations of leptin with body fat mass parameters, adjusting for potential confounders (age, BMI-SDS, and Tanner stage) and obtaining β coefficients and p-values. Finally, a simple linear regression analysis was performed to assess associations between LBP or chemerin levels and the number of MetS components after the 2- and 12-month interventions.

## 3. Results

### 3.1. Effect of Lifestyle Intervention

Baseline characteristics and changes in clinical parameters measured at 2 months (intensive phase) and 12 months of follow-up are detailed in Table 1. In the longitudinal analyses, after the 2 month intervention, BMI-SDS significantly decreased (−0.59 units, *p* < 0.001), as did most of the anthropometric parameters. DBP, lean mass and total body water did not change. After 10 months of follow-up, a significant decrease in BMI-SDS (−0.46 units, *p* < 0.001) was also observed.

In regard to biochemical parameters, total cholesterol, glucose, insulin and leptin levels were significantly decreased after the intervention program (2- and 12-months of follow-up) as seen in Table 1.

### 3.2. Effect of Lifestyle Intervention on Quemerin and LBP Plasma Levels

**Figure 1:**
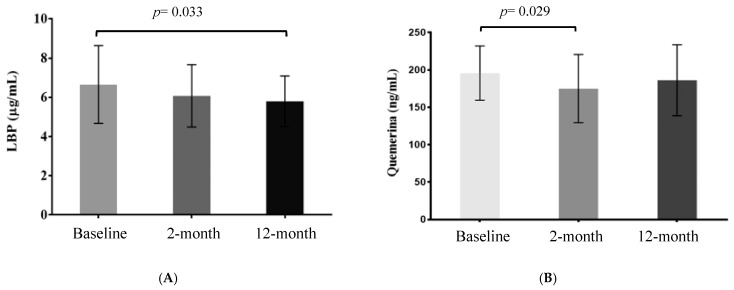
Lipopolysaccharide-binding protein (LBP) (**A**) and chemerin (**B**) plasma levels during the lifestyle intervention (baseline, 2 and 12 months) in pediatric patients with abdominal obesity. The ANOVA repeated measurements was performed followed by Tukey Post-Hoc Test.

LBP and chemerin plasma levels from pediatric patients with abdominal obesity are shown in Figure 1. There was a trend towards reduced values of these two markers throughout the lifestyle intervention (*p*-trend). When the repeated measures ANOVAs were performed followed by a *posteriori* test, LBP levels were significantly decreased between baseline and 12 months of follow-up (*p* = 0.033, Figure 1A), whereas a significant reduction in chemerin levels was observed between baseline and 2-month levels (Figure 1B, *p* = 0.029) in pediatric patients with abdominal obesity that followed the lifestyle intervention.

### 3.3. Associations between Adipokines and Metabolic Syndrome Features

As shown in Supplementary Figure S1, LBP plasma concentrations were significantly associated with leptin levels (Supplementary Figure S1A) and body fat mass (Supplementary Figure S1B). Chemerin plasma levels were associated with body fat mass (Supplementary Figure S1C) at baseline.

We analyzed the percentage of subjects with MetS [25] at baseline, month 2 and 12 (Table 2). After 12 months of lifestyle intervention, the number of pediatric subjects with MetS significantly decreased. Specifically, the number of subjects who presented WC greater than the 90th percentile (*p* ≤ 0.001) or a glucose level greater than 100 mg/dL (*p* = 0.030) was significantly lower as indicated in Table 2.

**Figure 2:**
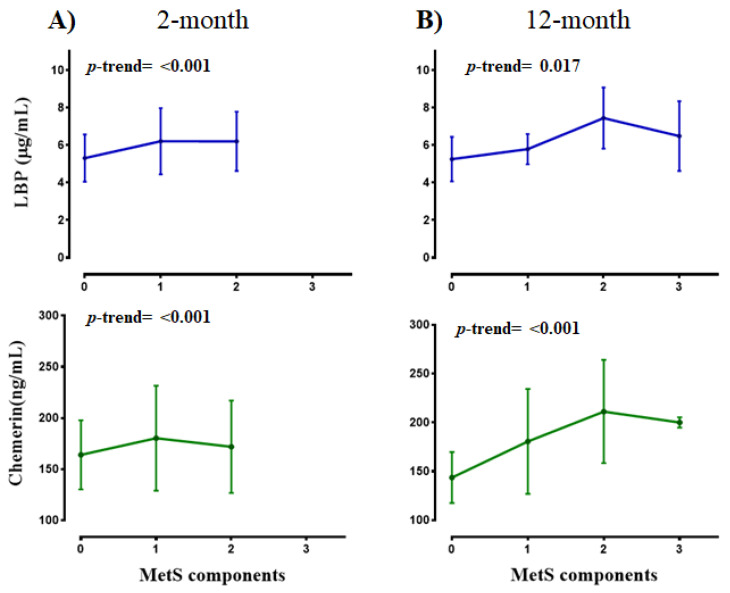
LBP and chemerin levels according to MetS components at 2 (**A**) and 12-month (**B**) of the lifestyle intervention in pediatric subjects with abdominal obesity. Data are mean ± SD. The p-trend test was applied.

Both LBP and chemerin plasma concentrations were analyzed in relation to MetS components, at different times (baseline, 2 and 12 months, Figure 2). Interestingly, higher values of both biomarkers were associated with a greater number of MetS components in this population.

## 4. Discussion

In this randomized controlled trial in pediatric subjects with abdominal obesity, a significant reduction in BMI-SDS and in MetS prevalence was observed after the two month intensive period as has been seen in other lifestyle intervention studies [26,27,28]. Some benefits of this lifestyle intervention were maintained at 12 months of follow-up. It is suggested that a decrease in cardiometabolic risk is achieved when the reduction in BMI-SDS is greater or equal to 0.2 [29]. In this regard, Reinehr et al. (2016) indicate that the decrease in BMI-SDS is linked to a reduction in both SBP and DBP [29]. However, in our study, neither systolic nor diastolic blood pressure decreased after 12 months of follow-up. Rajjo et al. (2016) reported an association between weight loss and the decrease in SBP [30], although Hvidt et al. (2014) found no relationship between changes in BMI-SDS and BP, thus supporting our results [31]. As for the glucose and lipid profiles, we observed a decrease in glucose, total cholesterol, and LDL-C levels after 2 and 12 months of follow-up in our pediatric population. These findings are similar to those reported in some pediatric intervention studies [28,32,33].

Weight loss is generally accompanied by changes in the profile of circulating adipokines. In particular, leptin levels decreased significantly after the lifestyle intervention in our population. This result is in line with the work of Siegrist et al. (2013). These authors found a significant reduction in leptin plasma levels after 1-year of a lifestyle intervention in adolescents with obesity [34].

Several cross-sectional studies have described higher LBP levels in children with obesity [14,35,36,37,38]. But, to our knowledge, there are no studies on LBP in lifestyle intervention programs. At baseline, we reported a significant association between LBP levels and leptin as found in other studies in obese subjects [3,36,38]. Moreover, we observed a significant reduction of LBP after 12 months of the intervention in pediatric subjects with abdominal obesity. A similar result was found in obese adults enrolled in a weight loss program [36].

In the present study, chemerin plasma levels decreased after 2 months of intervention in pediatric subjects with abdominal obesity. Similarly, Niklowitz et al. observed a decrease in chemerin plasma levels accompanied by a reduction in BMI-SDS after a 1-year intervention in German obese children [18]. In addition, in our intervention, we observed a significant association between the changes in leptin and chemerin levels after 2 months of the intervention. In line with this finding, Landgraf et al. (2012) observed an association between chemerin and leptin levels in obese children [9].

Our lifestyle intervention was able to reduce the metabolic risk of the pediatric population with abdominal obesity. In particular, a decrease in the prevalence of hyperglycemia, and abdominal obesity was noted. Similar to the work of Velázquez-López et al. (2014) in which a decrease in both BMI and MetS prevalence was observed after a 4 month-Mediterranean Diet intervention in Mexican children [38]. Interestingly, we observed an association between both LBP and chemerin levels and MetS components. In recent studies, both cytokines have been related to MetS determinants in several pediatric populations [37,39,40]. It is suggested that LBP could be a predictor of insulin resistance [37] and chemerin an early indicator of individuals at high metabolic risk (39). It is very interesting to discover biomarkers for MetS that could improve the early diagnosis and treatment in pediatric subjects.

The strengths of this study include the following: the longitudinal design, which provides the possibility of making paired comparisons with baseline data used as a control; the homogeneity of the group (similar number of boys and girls), the availability of plasma samples from participants at three time points; and that most participants achieved a significant reduction in BMI-SDS linked to a lower cardiovascular risk. The main weakness of this study is the limited sample size, which could influence our findings.

In conclusion, the present lifestyle intervention in a pediatric population with abdominal obesity was able to reduce some anthropometric and biochemical parameters. LBP levels decreased at 12 months of follow-up, whereas chemerin levels significantly fell after two months of intervention. In addition, these plasma biomarkers were associated with the prevalence of MetS. The use of biomarkers of MetS could increase the rate of early diagnosis and could prevent the complications of obesity. Nevertheless, large interventional studies in pediatric populations with obesity are needed to confirm our findings on chemerin and LPS as potential biomarkers for the progression of cardiovascular risk.

## Figures and Tables

**Table 1:** Anthropometric and biochemical variables pre- and post-lifestyle intervention in pediatric patients with abdominal obesity.

	At Baseline [*n* = 29]		2-Month [*n* = 29]			12-Month [*n* = 21]		
	Mean	SD	Mean	SD	*p* ^1^	Mean	SD	*p* ^2^
**Body weight [kg]**	69.24	21.72	66.14	20.81	**<0.001**	70.99	20.14	**<0.001**
**Sex [F/M]**			15/14		[52%/48%]			
**Height [cm]**	150.63	14.69	151.58	14.56	**<0.001**	155.28	14.67	**<0.001**
**BMI [kg/m^2^]**	29.67	5.21	27.96	4.98	**<0.001**	28.73	5.37	**<0.001**
**BMI-SDS**	3.05	1.09	2.46	1.11	**<0.001**	2.59	1.29	**<0.001**
**WC [cm]**	88.66	13.25	84.18	12.96	**<0.001**	85.15	13.23	**<0.001**
**WHR**	0.59	0.06	0.55	0.06	**<0.001**	0.55	0.06	**<0.001**
**HC [cm]**	101.12	14.28	98.35	13.99	**<0.001**	101.29	14.57	**<0.001**
**Fat mass [%]**	36.64	6.87	34.06	6.67	**<0.001**	33.91	7.68	**0.002**
**Fat mass [kg]**	26.29	11.85	23.34	10.55	**<0.001**	24.91	11.38	**<0.001**
**Lean mass [kg]**	43.02	11.82	42.83	11.97	0.514	46.11	11.61	**<0.001**
**Body water [kg]**	31.46	8.67	31.36	8.77	0.655	33.73	8.48	**<0.001**
**SBP [mmHg]**	119.66	12.45	113.38	14.35	**<0.001**	113.54	14.96	0.103
**DBP [mmHg]**	73.79	9.99	68.72	13.61	0.081	69.31	15.15	0.277
**Total-C [mg/dL]**	160.81	21.94	150.4	23.75	**0.007**	149.77	21.48	**0.009**
**LDL-C [mg/dL]**	98.24	17.69	92.27	20.36	**0.045**	88.60	18.55	**0.007**
**HDL-C [mg/dL]**	45.79	9.55	43.83	8.01	0.074	46.81	8.85	0.106
**Triglycerides [mg/dL]**	83.65	35.25	71.67	31.49	0.104	71.99	32.25	0.156
**Glucose [mg/dL]**	90.31	6.86	85.21	5.85	**<0.001**	88.34	6.39	**<0.001**
**Insulin [μU/mL]**	14.92	7.36	11.37	5.91	**0.003**	12.41	5.73	**0.005**
**Leptin [ng/mL]**	28.03	15.25	19.36	14.83	**0.003**	24.23	17.84	**0.012**

Abbreviations: SD, standard deviation; BMI, body mass index; BMI-SDS, BMI-standard deviation score; SBP, systolic blood pressure; DBP, diastolic blood pressure; HOMA-IR, homeostasis model assessment for insulin resistance; QUICKI, quantitative insulin sensitivity check index. *p*^1^: *p* values from paired *t*-test for the comparison between pre and post-intervention variables. *p*^2^: *p* values from repeated-measures ANOVA and Tukey Post-Hoc Test for the comparison between 3 different times. In bold *p* < 0.05.

**Table 2:** Prevalence of metabolic syndrome and its components in pediatric patients with abdominal obesity along the lifestyle intervention.

	Baseline [*n* = 29]	2-Months [*n* = 29]	12-Months [*n* = 21]	*p*
**MetS**	7 [24%]	0	4 [19%]	**0.009**
**MetS components**				
**WC ≥ 90th percentile**	29 [100%]	25 [86%]	17 [81%]	**<0.001**
**Triglycerides ≥ 150 mg/dL**	2 [7%]	1 [3%]	1 [5%]	0.779
**HDL-C ≤ 40 mg/dL**	9 [31%]	10 [35%]	5 [24%]	0.072
**Glucose ≥ 100 mg/dL**	5 [17%]	0 [0%]	1 [5%]	**0.030**
**SBP ≥ 130 mmHg**	8 [28%]	3 [10%]	3 [14%]	0.103
**DBP ≥ 85 mmHg**	6 [21%]	2 [7%]	2 [10%]	0.135

Abbreviations: MetS, metabolic syndrome; WC, waist circumference; HDL-C, HDL-cholesterol; LDL-C, LDL-cholesterol; SBP, systolic blood pressure; DBP, diastolic blood pressure. In bold *p* < 0.05 values obtained by applying the Cochran test. MetS definition—IDF criteria [25].

## Data Availability

Data supporting reported results will be available upon request.

## Supplementary Materials

The following are available online at https://www.mdpi.com/2072-6643/13/2/289/s1, Figure S1: Association between LBP and leptin (S1A), and between LBP (S1B) or chemerin (S1C) and body fat mass (S1B).
