# Supplementary material for: Higher Lipopolysaccharide Binding Protein and Chemerin Concentrations Were Associated with Metabolic Syndrome Features in Pediatric Subjects with Abdominal Obesity during a Lifestyle Intervention
Source: Nutrients. 2021 Jan 20;13(2):289. doi: 10.3390/nu13020289 (PMC7909441; doi:10.3390/nu13020289)
Supplement: Supplementary file 1 [file nutrients-13-00289-s001.pdf]

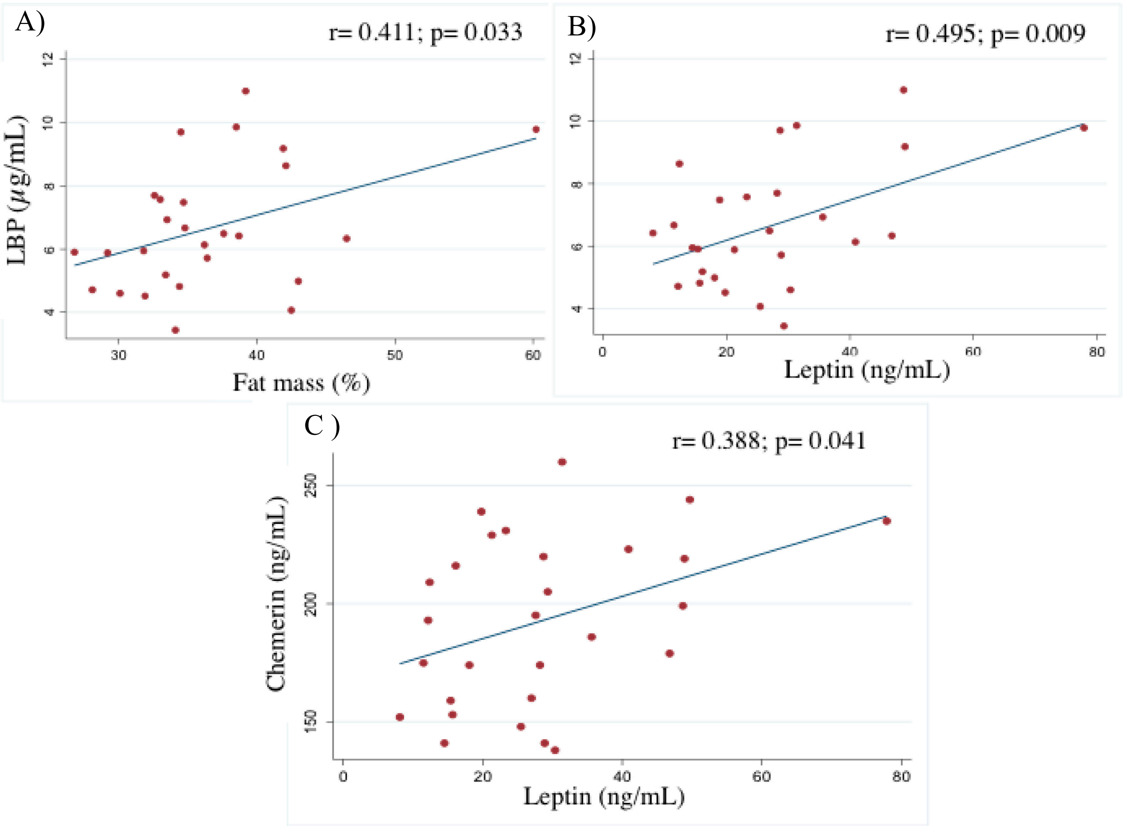

Figure S1. Correlation between LBP levels and fat mass [A] and leptin levels [B]. Correlation between chemerin and leptin levels [C].
